# Workforce mental health as health security infrastructure: protecting emergency response capacity during public health emergencies

**DOI:** 10.3389/fpubh.2026.1935035

**Published:** 2026-09-23

**Authors:** Abdullahi Ahmed Tahlil, Mohamed Abdelrahman Mohamed, Liban Abdi Ali, Mohamed Abdirahman Abdi, Hassan Sheikh Ahmed, Nur Ali Mohamud, Ali Haji Adam, Abdinasir Yusuf Osman

**Affiliations:** 1 Ministry of Health and Human Services, Federal Government of Somalia, Mogadishu, Somalia; 2 Department of Public Health, Faculty of Graduate Studies and Research, Somali National University, Mogadishu, Somalia; 3 Center for Epidemic Intelligence, Preparedness and Response, Mogadishu, Somalia; 4 Royal Veterinary College, University of London, London, United Kingdom

**Keywords:** burnout, emergency responders, health security, healthcare workers, mental health, moral injury, pandemic, PTSD

## Abstract

Public health emergencies place substantial psychological demands on healthcare and public health emergency response workforces. While preparedness frameworks traditionally emphasize surveillance systems, infrastructure, and operational capacity, workforce mental health is increasingly recognized as an important determinant of sustainable emergency response. This Perspective examines how workforce mental health functions as an underlying determinant of health security capacity. Evidence from contemporary literature indicates that emergency responders experience substantial burdens of psychological distress, including post-traumatic stress disorder (PTSD) symptoms, burnout, depression, anxiety, and moral distress. These outcomes are influenced by trauma exposure, organizational stressors, workload pressures, resource limitations, and ethical challenges encountered during emergency response. However, estimates vary across occupational groups, geographic settings, measurement approaches, and emergency contexts. This Perspective proposes that workforce mental health should be considered a measurable component of health security infrastructure. Protecting responder wellbeing requires a systems-based approach integrating organizational reforms, confidential support mechanisms, workforce protection policies, and evidence-informed interventions. Incorporating workforce mental health indicators into emergency preparedness frameworks may strengthen workforce sustainability, operational readiness, and health security resilience.

## 1 Introduction

Public health emergencies represent critical moments in which societies mobilize healthcare and emergency response workforces to protect population health and mitigate severe consequences. Whether stemming from infectious disease pandemics, natural disasters, mass casualty events, or bioterrorism threats, such crises impose demands fundamentally exceeding those encountered in routine operational environments (1). While considerable attention has focused on patient outcomes, disease transmission, and healthcare system capacity during public health emergencies, the psychological and emotional toll experienced by emergency response personnel has historically received comparatively less attention within preparedness frameworks, research priorities, and resource allocation strategies (1, 2). In this paper, the term healthcare and public health emergency response workforce refers primarily to personnel engaged in health-related emergency preparedness and response activities, including emergency physicians, nurses, paramedics, public health professionals, epidemiologists, laboratory personnel, and other frontline health workers. These groups experience overlapping but distinct occupational exposures, and differences in professional roles, working environments, organizational structures, and health system contexts should be considered when interpreting evidence (2, 3).

The emergence of the COVID-19 pandemic has forcefully redirected attention toward an unfolding mental health crisis within emergency response workforces. Frontline healthcare workers experienced unprecedented occupational demands accompanied by exposure to communicable disease, moral distress stemming from resource scarcity and inability to provide optimal care, pervasive social isolation, and societal criticism despite extraordinary sacrifice (2, 3). During the acute COVID-19 pandemic response, studies on emergency physicians documented that 85% of respondents experienced negative psychological effects (4). However, the mental health burden of emergency responders extends well beyond COVID-19, reflecting chronic vulnerability inherent to emergency response work itself. Historically, workforce mental health has received limited attention due to multiple factors, including occupational cultures emphasizing resilience and stoicism, stigma surrounding psychological vulnerability, prioritization of physical safety risks over psychological hazards, and limited investment in occupational mental health research and services. These gaps have contributed to the under-recognition of mental health as an essential component of emergency preparedness. The evidence was interpreted across three temporal contexts: pre-COVID-19 emergency response literature describing longstanding occupational risks; COVID-19 pandemic-era evidence demonstrating crisis-related amplification of mental health burdens; and emerging post-pandemic literature addressing persistent workforce and health system challenges.

This Perspective advances the argument that workforce mental health should be considered a measurable component of health security infrastructure. Rather than viewing responder mental health as an individual occupational wellbeing issue, we propose a systems framework linking psychological wellbeing, workforce sustainability, emergency readiness, and health security performance. We synthesize evidence from contemporary peer-reviewed literature to elucidate the scope of this challenge, explore pathways through which workforce mental health may influence emergency response capability, identify evidence-based protective factors and interventions, and present integrated recommendations for protective intervention at individual, organizational, and health system levels.

## 2 Approach to evidence identification and synthesis

This Perspective article was developed using a structured narrative synthesis approach to identify and integrate contemporary evidence regarding mental health risks among emergency response workforces and their implications for health security. A targeted search of peer-reviewed literature was conducted using PubMed/MEDLINE, Scopus, Web of Science, and Google Scholar. Searches focused on publications available between January 2010 and March 2026 using combinations of keywords including “emergency responders,” “healthcare workers,” “public health emergencies,” “PTSD,” “burnout,” “moral injury,” “psychological distress,” “workforce resilience,” and “health security.”

Evidence was selected based on relevance to emergency response occupations, methodological rigor, and contribution to understanding mental health outcomes, organizational determinants, workforce consequences, or intervention approaches. Priority was given to systematic reviews, meta-analyses, longitudinal studies, and large observational studies. Because this article represents a Perspective rather than a systematic review, formal risk-of-bias assessment and quantitative meta-analysis were not undertaken. Instead, evidence was critically interpreted to develop a conceptual framework linking workforce mental health with emergency preparedness and health security capacity.

## 3 Mental health outcomes and occupational psychological stressors among emergency response workforce

This paper distinguishes between psychiatric conditions, occupational stress syndromes, and emerging psychological constructs. PTSD, depression, and anxiety represent clinically recognized mental health conditions. Burnout is an occupational phenomenon characterized by emotional exhaustion, depersonalization, and reduced professional efficacy resulting from chronic workplace stress. Moral distress and moral injury represent related but distinct experiences involving ethical conflicts, perceived violations of deeply held values, or inability to provide care consistent with professional expectations. Although these concepts may overlap and influence one another, they should not be considered interchangeable.

### 3.1 Prevalence and burden of PTSD, burnout, and psychological distress

Mental health disorders and psychological distress are highly prevalent within emergency response occupations, with prevalence rates substantially exceeding general population prevalence. A systematic review and meta-analysis examining emergency department personnel in North America estimated that 18.6% of emergency care workers met criteria for PTSD (5). Although PTSD symptoms and probable PTSD identified through screening instruments appear common among emergency response personnel, comparisons with general population estimates should be interpreted cautiously. Differences in diagnostic methods, screening thresholds, study populations, exposure characteristics, and assessment periods limit direct comparisons across studies.

Burnout, operationalized as a syndrome encompassing emotional exhaustion, depersonalization, and diminished personal accomplishment (6), affects substantial proportions of emergency response personnel across diverse geographic settings. Studies conducted among emergency medical services personnel, emergency department nurses and physicians, and paramedics across diverse settings, including North America, Europe, and other healthcare systems, have reported burnout prevalence ranging from 62 to 86%; however, estimates vary according to occupational group, country context, measurement instruments, and study design (7–9). During the acute phase of the COVID-19 pandemic, 66.2% of public health professionals reported clinically significant burnout, with profound implications for workforce retention (10). Among public health workers surveyed, 23.6% fewer respondents intended to remain within the public health workforce for a minimum of 3 years compared to their pre-pandemic employment intentions, reflecting substantial erosion of workforce commitment during the crisis phase (10).

### 3.2 Suicide and mortality risks

Particularly concerning are suicide rates among healthcare workers. Suicide-related outcomes among healthcare and emergency response workers represent an important occupational mental health concern (11). Available evidence suggests variation in suicidal ideation and suicide risk across professional groups and settings, with some occupational cohorts demonstrating increased vulnerability compared with reference populations (12–14). However, comparisons require careful interpretation because suicide estimates are influenced by age, sex distribution, occupational category, healthcare system context, observation period, and measurement methods.

## 4 COVID-19 pandemic magnification

The COVID-19 pandemic substantially intensified pre-existing mental health vulnerabilities. During the pandemic response, 85% of emergency physicians reported negative mental health impacts, including anxiety, depression, insomnia, and PTSD, with daily alcohol use nearly doubling compared with pre-pandemic baseline rates (14). A survey of 924 COVID-19 frontline healthcare workers in Iran documented that emotional exhaustion constituted the primary mediator of diminished mental health, highlighting the importance of occupational workload and psychological stressors within the specific healthcare context studied (10). Among nurses working in emergency departments and intensive care units, the pandemic was associated with significantly elevated burnout scores, with working in the emergency room identified as an independent risk factor for moderate-to-severe mood disorder (adjusted odds ratio, 2.81; 95% CI, 1.14–6.90) (15). Longitudinal assessment of healthcare professionals during the pandemic revealed that demographic characteristics substantially modulated mental health outcomes. Younger age, female gender, greater COVID-19 exposure, lower baseline resilience, and immature defensive functioning predicted elevated stress and burnout (*R*^2^ = 0.463; *p* < 0.001) (16). These differential impacts suggest that existing health disparities within healthcare systems become magnified during crises, with vulnerable worker populations bearing disproportionate mental health burdens. Beyond measurable mental health outcomes, emergency response environments may generate specific psychological experiences related to ethical conflicts and perceived inability to provide appropriate care.

## 5 Moral injury and ethical distress: emerging mechanisms

Beyond trauma exposure and burnout, contemporary research illuminates moral injury as a distinct psychological mechanism contributing substantially to mental health deterioration among emergency response workers. Moral injury, defined as psychological damage arising when individuals transgress or witness transgressions of deeply held moral values, or when institutions with a duty to provide care fail to do so, emerged as a dominant mental health concern during the COVID-19 pandemic (17). Recent studies suggest that moral injury and moral distress are associated with psychological outcomes including PTSD symptoms, depression, anxiety, and burnout among healthcare workers (18). However, the magnitude of these associations varies across populations and measurement approaches, and further research is needed to clarify causal pathways (19, 20).

Psychological manifestations of moral injury include incapacitating feelings of guilt and shame, anxiety, depression, post-traumatic stress disorder symptoms, and elevated suicidality (17, 21). Beyond depression and anxiety, moral injury generates behavioral outcomes including disturbances in sleep and reduced treatment-seeking behavior (21). The pandemic context particularly catalyzed moral injury through impossible resource allocation decisions, inadequate personal protective equipment, inability to provide dignified end-of-life care, and inadequate recognition for extraordinary sacrifice. Moral injury and moral distress may each independently predict PTSD symptoms; however, their combined effect substantially amplifies psychological harm. Understanding these outcomes requires consideration of the occupational and organizational pathways through which emergency response demands translate into psychological burden.

## 6 Potential pathways linking emergency response demands to mental health outcomes

The pathways described below represent evidence-informed and proposed pathways through which emergency response demands may contribute to mental health outcomes. While some associations are supported by empirical evidence, the relative contribution of specific pathways may vary across occupational groups, settings, and mental health outcomes.

### 6.1 Cumulative traumatic exposure

Emergency response personnel experience repeated, often extreme exposure to aversive circumstances, including serious injury, death, suffering, and human tragedy. The cumulative exposure process is often non-volitional and progressive throughout careers; unlike civilians experiencing circumscribed traumatic events, responders encounter successive trauma throughout professional tenure, which may contribute to reduced psychological resilience and increased vulnerability to traumatic stress responses.

### 6.2 Organizational and systemic stressors

Qualitative research examining ambulance personnel reveals that responders perceive minimal organizational support for mental health concerns, widespread stigmatization of mental illness within emergency services cultures, and reluctance to seek professional mental health services due to anticipated career consequences (22). Understaffing, excessive workload, inadequate resources, and perception of organizational undervaluation contribute substantially to burnout. The lack of acknowledgement or recognition of responder contributions constitutes a specific risk factor for mental health deterioration (23). During the COVID-19 pandemic, public health professionals faced intense public hostility, harassment, and job-related threats. Surveys of local health departments showed that over half of the responding offices reported harassment incidents targeting officials, leading many leaders to experience severe psychological distress and step down from their roles (24). A study comparing health worker experiences between 2018 and 2022 revealed that bullying, harassment, and threats from patients and coworkers doubled, burnout increased from 32 to 46%, and harassed workers experienced 5-fold greater anxiety, 3-fold greater depression, and 6-fold greater burnout than non-harassed workers (25). These findings demonstrate that the organizational environment substantially modulates mental health trajectories independently of clinical workload.

### 6.3 Occupational culture and help-seeking barriers

Emergency response organizations have historically cultivated cultures emphasizing stoicism, self-reliance, and minimization of psychological vulnerability. Ambulance personnel report that while organizations provide peer support programs, actual helpful debriefing occurs largely outside formal organizational channels, with family or friends rather than professional resources (22). Seventy-five percent of emergency department healthcare workers endorsing clinically significant psychiatric symptoms during the pandemic did not seek formal psychological support, indicating substantial unmet mental health treatment needs despite symptom severity (26). State medical board licensing requirements requiring disclosure of mental health conditions contribute to help-seeking avoidance among physicians, who face additional scrutiny under assumptions of incompetence (25). Occupational stigma prevents help-seeking; physicians receive training in “medical exceptionalism” and self-negation that promotes burnout and discourages disclosure of work-related mental injury.

The psychological consequences described above extend beyond individual wellbeing and may influence the operational functioning of emergency response systems. Understanding these implications requires consideration of how workforce mental health interacts with patient safety, workforce sustainability, and broader health security capacity.

## 7 Implications for health security, patient safety, and organizational capacity

### 7.1 Cognitive and operational impairment

Mental health challenges among emergency response workforces may influence health security capacity through potential pathways involving workforce availability, cognitive functioning, decision-making processes, and organizational performance. Post-traumatic stress disorder, burnout, depression, and anxiety among emergency responders have been associated with potential impairments in judgment, cognitive functioning, decision-making, and situational awareness in high-stakes emergency contexts. Research indicates that PTSD symptoms among emergency personnel are associated with reduced quality of patient care indicators, increased lost workdays, elevated staff turnover, and challenges in maintaining emergency response capability (27). Burnout among healthcare workers has been associated with increased patient safety concerns, reduced quality of care indicators, and impaired professional functioning (28).

### 7.2 Workforce retention and organizational sustainability

An important consequence of workforce mental health deterioration is accelerated workforce attrition. The already understaffed public health workforce faces substantial retention challenges exacerbated by pandemic-related burnout. Approximately 80,000 additional full-time staff members are required throughout the nation’s public health agencies to meet current service needs (29). This deficit may be further exacerbated by workforce losses associated with burnout and mental health challenges. Workforce mental health challenges may contribute to turnover intention, absenteeism, and workforce instability among healthcare professionals. Loss of experienced personnel may reduce institutional knowledge, increase workload among remaining staff, and weaken emergency response sustainability (28). Emergency medical services, emergency departments, and critical care units similarly confront workforce shortages amplified by burnout-driven attrition.

### 7.3 Economic impact and healthcare system strain

Burnout imposes substantial economic consequences beyond direct turnover costs. Emotional exhaustion contributes substantially to turnover intention, reducing discretionary effort and willingness to work additional shifts, undermining staffing resilience precisely when surge capacity becomes essential (30). Burnout also suppresses institutional commitment, with 14% of burned-out clinicians reporting contemplation of quitting medicine during individual shifts (28). In many settings, public health funding mechanisms may result in mental health programs relying on short-term or unstable financing streams rather than sustained institutional investment. Although COVID-19 emergency funding provided temporary support for public health and mental health programs, many systems continue to face broader structural challenges, including unstable financing, workforce shortages, competing health priorities, and limited investment in sustainable occupational mental health infrastructure (31). This funding withdrawal occurred simultaneously with sustained elevated emergency response demands, creating an untenable mismatch between workforce needs and available support. While emergency response environments create substantial risks, evidence also identifies individual, social, and organizational factors that may protect workforce mental health.

## 8 Protective factors and evidence-based interventions

### 8.1 Individual, organizational, and structural protective approaches

Resilience refers to the dynamic capacity of individuals and systems to adapt successfully to stressors while maintaining psychological functioning and occupational effectiveness (32). Longitudinal studies on healthcare workers and emergency-related populations show that higher baseline resilience acts as a strong protective buffer, leading to fewer post-traumatic stress, anxiety, and depression symptoms following high-stress critical incidents. Resilient individuals handle acute strain better and display stable functioning over time (33). Specialized training, elevated levels of professional mastery, confidence in personal and team capabilities, and satisfaction with professional role each independently predict reduced stress and lower PTSD risk (34). Evidence for peer-support interventions is strongest for improving perceived support, reducing stigma, and increasing willingness to seek help. Evidence for direct effects on specific psychiatric outcomes such as PTSD, depression, or suicidality remains more limited and should be interpreted cautiously.

Importantly, resilience is not a fixed trait; evidence indicates that resilience training has demonstrated potential benefits in improving psychological outcomes, although effectiveness varies depending on intervention design, population characteristics, and outcome measures. A study reported beneficial effects of resilience training interventions, including digital formats, on stress and burnout outcomes among healthcare workers; however, effect sizes vary across populations and intervention designs (35). Multi-component interventions incorporating psychoeducation, mindfulness, and stress management components have demonstrated potential benefits for improving resilience-related outcomes among healthcare workers (36). Notably, resilience training does not generate harm in the form of psychological distress or memory impairment, unlike critical incident stress debriefing interventions that some personnel report as intrusive and distressing. Importantly, resilience-oriented approaches should not be interpreted as placing responsibility for occupational mental health solely on individual workers. Psychological outcomes among emergency responders are strongly influenced by organizational conditions, including staffing levels, workload intensity, leadership practices, workplace safety, resource availability, and institutional culture. Therefore, individual resilience interventions should complement, rather than replace, structural reforms aimed at reducing occupational hazards and improving working environments.

Comparative effectiveness analysis reveals that positive psychology (e.g., psychological capital training) demonstrates the largest improvements in resilience, followed by mindfulness-based stress reduction and cognitive behavioral therapy approaches, with sustained effects observed (37). Anger management psychoeducation, emotional intelligence training, and mindfulness-based stress reduction programs demonstrated the most promising effects for improving resilience in nurses, with network meta-analysis demonstrating offline field interventions outperforming online interventions (38). Digital resilience interventions have shown promising effects, although evidence remains heterogeneous (39).

### 8.2 Workforce support and peer-based interventions

Social integration, peer support, and positive team cohesion emerge consistently as buffers against PTSD development among disaster responders and emergency workers (33). Professional peer support programs integrated into first responder organizational culture appear particularly promising, as they leverage trusted peer relationships to reduce treatment-seeking stigma that impedes formal mental health service utilization (40). Mature defensive functioning and adaptive coping mechanisms predict resilience and personal accomplishment, while neurotic and immature defenses correlate with higher stress and burnout (16). Frontline clinicians reported substantial benefit from peer-to-peer support and access to dedicated wellness spaces during the pandemic (41). Patient safety culture improvements, including team cohesion, effective handoffs, and transitions, demonstrate associations with reduced workplace violence and burnout among health care workers (42). Organizations implementing “Magnet hospital” models, institution-wide interventions promoting positive work environments, demonstrate improved workforce wellbeing and retention (43). Organizations must prioritize creating and promoting supportive, healthier workplace cultures if they want to retain a thriving workforce (44).

### 8.3 Psychological support and organizational policy interventions

Intervention studies suggest that both individual-focused and organizational approaches may improve burnout-related outcomes. However, effects vary considerably, and organizational interventions addressing workload, staffing, leadership, and workplace conditions may have greater potential for sustained impact than interventions focused solely on individual coping (45). Organizational policy reforms addressing workload management, scheduling practices, resource adequacy, and workplace recognition have been associated with measurable improvements in worker psychological wellbeing. Duration of employment and lower job dissatisfaction act as protective and risk factors, respectively, against PTSD and burnout (23).

The Centers for Disease Control and Prevention and the National Institute for Occupational Safety and Health established a comprehensive five-part plan to raise awareness of mental health concerns among healthcare workers and foster sustainable infrastructure supporting future healthcare workforce wellbeing (44). This framework encompasses primary prevention through awareness and occupational stress reduction, secondary prevention through early identification of mental health symptoms, and tertiary prevention through treatment accessibility and organizational support for treatment engagement. Primary prevention approaches emphasizing sleep, exercise, peer engagement, and meditation require organizational structures facilitating such practices despite high-demand operational contexts.

## 9 Barriers to implementation and systemic obstacles

Despite clear evidence establishing the necessity of comprehensive mental health support for emergency response workforces and demonstrated efficacy of intervention approaches, substantial barriers impede implementation. The occupational culture of emergency services has historically emphasized emotional restraint and psychological self-reliance, creating environments where mental health vulnerability is perceived as occupational failure (44). Cultural expectations emphasizing emotional restraint and self-reliance mean that the individual who persists without complaint through extraordinary hardship remains culturally valorized in ways that paradoxically undermine actual wellbeing and treatment-seeking.

Resource limits are a major barrier to mental health care. Many low- and middle-income countries face substantial workforce shortages, limited mental health services, and constrained public health systems. These critical gaps hurt entire communities, not just people with mental disorders (46). Financially constrained emergency response organizations lack resources for mental health frameworks due to fluctuating public health budgets and external funding. Low-income nations face a severe deficit in formal psychological services. This shortage places an unsustainable burden on an exhausted healthcare workforce (47).

Additionally, the predominant biomedical focus of healthcare systems often relegates occupational mental health to peripheral importance relative to clinical operations. The reframing of workforce mental health as essential infrastructure rather than a peripheral employee benefit represents a necessary philosophical shift that has yet to achieve widespread institutional adoption. Without such reframing, mental health remains vulnerable to deprioritization when organizational resources become constrained.

## 10 Implementation risks and ethical considerations

Although workforce mental health interventions are increasingly recognized as essential components of emergency preparedness, implementation requires careful consideration of potential unintended consequences. Mandatory psychological interventions may create resistance or undermine trust if workers perceive them as organizational surveillance rather than support. Similarly, psychological screening programs require appropriate confidentiality protections to prevent stigma, discrimination, or professional consequences associated with disclosure. Digital mental health interventions may expand access, particularly in resource-limited settings; however, issues related to data security, privacy, digital literacy, and appropriate clinical supervision must be addressed. Peer-support programs also require structured training, clear boundaries, referral pathways, and ongoing supervision to ensure safety and effectiveness.

## 11 Workforce mental health in low- and middle-income countries

The mental health challenges experienced by emergency response workers may be particularly pronounced in low- and middle-income countries (LMICs), where emergency responders frequently operate within resource-constrained environments characterized by workforce shortages, limited mental health services, fragile infrastructure, conflict, displacement, and repeated humanitarian crises. These challenges are particularly relevant in fragile and conflict-affected settings, where emergency response workforces frequently operate under prolonged exposure to crises while mental health services remain limited. Furthermore, available evidence from LMIC settings is heterogeneous, reflecting differences in conflict exposure, healthcare system capacity, workforce availability, cultural perceptions of mental health, and access to occupational support services. Therefore, interventions developed in high-income settings require contextual adaptation before implementation in resource-constrained environments. Future approaches should avoid transferring intervention models from high-income settings without adaptation and should consider local health system capacity, cultural context, workforce availability, and feasibility.

## 12 A conceptual framework: workforce mental health as health security infrastructure

We propose a conceptual framework in which workforce mental health functions as a foundational determinant of emergency response capacity. Emergency exposure and organizational stressors, including traumatic events, workload intensity, resource limitations, ethical conflicts, and workplace insecurity, may contribute to psychological outcomes such as burnout, PTSD symptoms, anxiety, depression, and moral distress. These outcomes may influence health security through intermediate mechanisms including absenteeism, reduced workforce retention, impaired operational readiness, decreased surge capacity, and reduced institutional resilience during emergencies. To operationalize workforce mental health as a measurable component of health security, we propose a set of indicators spanning workforce wellbeing, sustainability, emergency readiness, organizational support, health security performance, and governance integration (Table 1).

**Table 1:** Proposed indicators for integrating workforce mental health into health security frameworks.

Framework domain	Potential indicators
Workforce mental health status	Burnout prevalence, psychological distress screening results, PTSD symptom burden
Workforce sustainability	Staff turnover intention, absenteeism, retention rates
Emergency readiness	Availability of trained responders, surge capacity, deployment readiness
Organizational support	Availability of confidential psychological services, peer-support mechanisms, occupational health programs
Health security performance	Response capacity, workforce availability during emergencies, continuity of essential services
Governance and policy integration	Inclusion of workforce mental health within national preparedness plans and emergency response policies

These indicators may support future operationalization of workforce mental health as a measurable component of health security. Their adaptation should consider national context, health system capacity, and available resources. Addressing the mental health burden of emergency response workforces requires fundamental reconceptualization of occupational mental health from a peripheral employee wellness concern to a critical component of public health emergency preparedness and health security infrastructure. This reframing carries important implications for resource allocation, organizational prioritization, and policy development. Health security encompasses not only the absence of infectious disease threats but also the maintenance of organizational capacity to detect, respond to, and mitigate health emergencies. Emergency response workforce wellbeing represents an important component of health security capacity. A health security architecture that does not incorporate workforce psychological wellbeing may face challenges in sustaining effective emergency response capacity. Therefore, workforce mental health support should be integrated within emergency preparedness planning, funded through biosecurity appropriations rather than competing with other occupational safety investments, and evaluated using health security metrics rather than peripheral employee wellness measures.

This framework necessitates coordinated action across multiple governance levels and stakeholders. Organizational leadership must establish workplace cultures that integrate mental health support into operational planning and resource allocation. Occupational health and safety infrastructure must be expanded to encompass psychological hazard assessment and control comparable to physical hazard frameworks. Public health agencies must develop population-level approaches to occupational mental health comparable to current approaches to occupational physical health and safety. National and international health security policy must incorporate workforce mental health sustainability within emergency preparedness protocols, establishing baseline funding, training requirements, and performance metrics.

## 13 Conclusion

The mental health burden associated with public health emergencies among emergency response workforces represents a substantial yet historically under-recognized challenge for health security capacity, patient safety, and organizational sustainability. Emergency responders experience substantial reported burdens of PTSD symptoms, burnout, depression, anxiety, and suicidal ideation, although estimates vary across occupational groups and settings. These findings highlight the importance of integrating workforce mental health considerations into emergency preparedness and health security frameworks. Mental health challenges among emergency response workers may influence workforce retention, quality of emergency care, cognitive functioning, decision-making capacity, and overall organizational resilience.

The COVID-19 pandemic illuminated the urgency of this challenge while revealing systemic vulnerabilities in mental health support infrastructure. Protective factors and evidence-based interventions exist to mitigate the mental health burden through the development of individual resilience, the integration of peer support, organizational policy reform, and health system restructuring. However, implementation remains constrained by occupational culture legacies, resource limitations, and failure to conceptualize workforce mental health as essential health security infrastructure. Addressing this challenge requires a fundamental reorientation of priorities, substantial resource reallocation, and the integration of occupational mental health into emergency preparedness frameworks.

## Data Availability

Publicly available datasets were analyzed in this study. This data can be found here: No datasets were generated or analyzed during the preparation of this manuscript. All information discussed is based on publicly available published literature, which is appropriately cited within the manuscript.
